# Multiple rearrangements and low inter- and intra-species mitogenome sequence variation in the *Heterobasidion annosum* s.l. species complex

**DOI:** 10.3389/fmicb.2023.1159811

**Published:** 2023-05-18

**Authors:** Kajsa Himmelstrand, Mikael Brandström Durling, Magnus Karlsson, Jan Stenlid, Åke Olson

**Affiliations:** Uppsala BioCenter, Department of Forest Mycology and Plant Pathology, Swedish University of Agricultural Sciences, Uppsala, Sweden

**Keywords:** comparative mitochondrial genomics, rearrangements, homing endonucleases, introns, accessory genes, optional GC palindromes, partially duplications

## Abstract

**Introduction:**

Mitochondria are essential organelles in the eukaryotic cells and responsible for the energy production but are also involved in many other functions including virulence of some fungal species. Although the evolution of fungal mitogenomes have been studied at some taxonomic levels there are still many things to be learned from studies of closely related species.

**Methods:**

In this study, we have analyzed 60 mitogenomes in the five species of the *Heterobasidion annosum sensu* lato complex that all are necrotrophic pathogens on conifers.

**Results and Discussion:**

Compared to other fungal genera the genomic and genetic variation between and within species in the complex was low except for multiple rearrangements. Several translocations of large blocks with core genes have occurred between the five species and rearrangements were frequent in intergenic areas. Mitogenome lengths ranged between 108 878 to 116 176 bp, mostly as a result of intron variation. There was a high degree of homology of introns, homing endonuclease genes, and intergenic ORFs among the five *Heterobasidion* species. Three intergenic ORFs with unknown function (uORF6, uORF8 and uORF9) were found in all five species and was located in conserved synteny blocks. A 13 bp long GC-containing self-complementary palindrome was discovered in many places in the five species that were optional in presence/absence. The within species variation is very low, among 48 *H. parviporum* mitogenomes, there was only one single intron exchange, and SNP frequency was 0.28% and indel frequency 0.043%. The overall low variation in the *Heterobasidion annosum sensu* lato complex suggests a slow evolution of the mitogenome.

## Introduction

Mitochondria are organelles present in almost all eukaryotic cells and originated from an α-proteobacterium that was engulfed by the ancestor of eukaryotes (Gray et al., 2001; Bullerwell and Lang, 2005; Koumandou et al., 2013). Since then, its genome has gone through a massive reduction in length and reshuffling in all eukaryotic lineages. The resulting double membrane organelle produces energy in the form of ATP with the help of electron transport and oxidative phosphorylation and performs other important functions in metabolism, ion homeostasis, and apoptosis (Burger et al., 2003). Mitochondria have also been shown to be important for the virulence of many fungal species (Olson and Stenlid, 2001; Shingu-Vazquez and Traven, 2011; Chatre and Ricchetti, 2014; Calderone et al., 2015; Medina et al., 2020). Most mitochondrial proteins are encoded by genes localized in the nuclear genome, but some proteins are encoded by genes localized in the mitochondrial genome (mitogenome) (Adams and Palmer, 2003) and fungal fitness is influenced by a close interplay by these nuclear and mitochondrial encoded proteins (Giordano et al., 2018; Clergeot and Olson, 2021).

Genes of fungal mitogenomes can be classified into two types: core genes and accessory genes. There are 15 core protein-coding genes (*nad1, nad2, nad3, nad4, nad4L, nad5, nad6, cob, cox1, cox2, cox3, atp6, atp8, atp9,* and *rps3*) and two RNA genes (*rns* and *rnl*), with some exceptions, in all phyla (Adams and Palmer, 2003; Fonseca et al., 2021) with functions in respiration, oxidative phosphorylation, and translation (Burger et al., 2003). On the other hand, the accessory open-reading frames (ORFs) or genes vary greatly in types, numbers, and positions (Himmelstrand et al., 2014; Araújo et al., 2021; Fonseca et al., 2021). Accessory genes are located in the introns of core genes (intronic ORFs) or in regions between the core genes (intergenic ORFs). Some of the intergenic ORFs descend from mitochondrial plasmids that have been integrated into the mitogenomes (Yui et al., 1988; Cahan and Kennell, 2005; Wang et al., 2008; Ferandon et al., 2013; Himmelstrand et al., 2014), whereas other intergenic ORFs have unknown function and origin and are typically not conserved between species (Araújo et al., 2021; Fonseca et al., 2021). However, some links between the plasmid-derived ORFs and the non-conserved ORFs can be found which suggest that they, in some cases, have the same origin (Himmelstrand et al., 2014). It is common that the introns of the core genes have conserved RNA secondary structures involved in autocatalytic splicing and the introns often contain ORFs coding for homing endonuclease genes (HEGs) (Lang et al., 2007; Hausner et al., 2014). The HEG-encoded enzyme cleave DNA at rare recognition sites and the introns can be inserted into genes by two different mechanisms depending on the intron type (group I or II) (Stoddard, 2005). This movement of introns is called intron homing, and horizontal transfer of introns occurs regularly, thereby contributing to the high intron and intronic ORF variation (Burt and Koufopanou, 2004; Stoddard, 2005). Variations in the presence/absence of introns and intergenic regions and their accessory ORFs, and in some cases repeat elements, contribute to the large length differences in fungal mitogenomes (Burger et al., 2003; Himmelstrand et al., 2014; Araújo et al., 2021; Fonseca et al., 2021).

The recent advancements in sequencing techniques have generated large amounts of information on mitogenomes. At present, there are more than 11,000 animal, 900 fungal, and 400 plant mitogenomes on the Organelle Genome Resources at NCBI.^1^ Most of the sequenced fungal mitogenomes are from the two Ascomycete classes *Saccharomycetes* and *Sordariomycetes* and the Basidiomycete class *Agaricomycetes*. The majority of comparative studies of fungal mitogenomes have earlier been focused on higher-order taxonomic levels, but in recent times, more closely related species have been studied. Comparative studies between closely related species within the same genus and even isolates within the same species provide excellent opportunities to gain a deeper understanding of the mechanisms that drive the evolution of mitogenomes.

The mitogenome lengths vary considerably between fungal species, ranging from 332,165 bp in *Golovinomyces cichoracearum*^2^ to 12,055 bp in *Rozella allomycis* (James et al., 2013). Even within genera, there can be a large variation in mitogenome length, e.g., in the ascomycete *Chrysoporthe* (Kanzi et al., 2016) and in basidiomycetes, *Pyrrhoderma*, *Amanita,* and *Ganoderma* genera (Lee et al., 2019; Li et al., 2020a, 2022b). In ascomycetes, the length variation is typically associated with introns, while the proportion of intergenic areas and intergenic ORFs is often relatively small (Friedrich et al., 2012; Joardar et al., 2012; Sulo et al., 2017; Brankovics et al., 2020; Kwak, 2020; Fonseca et al., 2021). In basidiomycetes of the Agaricomycete class, it is the rule rather than the exception that both introns and intergenic areas contribute to variation in mitogenome lengths and the lengths of intergenic areas and intergenic ORFs are often relatively large (Li et al., 2018, 2019a, 2019c, 2020a, 2021, 2022b). Plasmid-derived sequences and putative DNA polymerase genes are present in all investigated Agaricomycete genera (Himmelstrand et al., 2014).

Fungal mitogenomes are reported to have lower substitution rates compared with animal mitogenomes. Synteny is nevertheless low at higher fungal taxonomic levels and in the basidiomycete phylum in particular (Aguileta et al., 2014; Ye et al., 2020). However, exceptions are found in the ascomycete classes Sordariomycetes and Eurotiomycetes, and there is often a complete synteny of the core genes within genera in the classes Leotiomycetes and Saccharomycetes (Friedrich et al., 2012; Joardar et al., 2012; Torriani et al., 2014; Kanzi et al., 2016; Zhang et al., 2017b). On the other hand, in Agaricomycetes, rearrangements of core genes are common within genera and core genes can be positioned on both strands (Himmelstrand et al., 2014; Li et al., 2019b, 2020a, 2020b; Chen et al., 2021; Wu et al., 2021). Frequent rearrangements of intergenic regions and intergenic ORFs are common (Li et al., 2018, 2019a, 2019c), sometimes resulting in considerable mitogenome length differences within genera (Lee et al., 2019; Li et al., 2019c).

A number of comparative studies of mitogenomes within species have been carried out in Ascomycetes (Torriani et al., 2008; Joardar et al., 2012; Jung et al., 2012; Bartelli et al., 2013; Freel et al., 2014; Wolters et al., 2015; Zhang et al., 2015, 2017a; Wang et al., 2018; Kulik et al., 2020; Yang et al., 2020). In these within-species studies, there were no large rearrangements, and the variation found in mitogenome lengths was due to introns and/or intergenic regions. The length difference ranged from 355 bp between two *Tolypocladium inflatum* isolates (Zhang et al., 2017a) to 15,000 bp in a comparison of six *Annulohypoxylon stygium* isolates (Deng et al., 2018).

Limited information is available concerning within-species variation of mitogenome length and structure in Agaricomycetes. There are 48 reference assembled genomes of *Hypsizygus marmoreus* reported by Wang et al. (2019), and 59 *de novo-*assembled mitogenomes of *Pyrrhoderma noxium* (previously *Phellinus noxius*) isolates have been studied by Lee et al. (2019). In *P. noxium,* a strong correlation was found between mitogenome length and intergenic region lengths (Lee et al., 2019). Rearrangements in intergenic regions between isolates were observed with a strong association between repetitive regions and rearrangement breakpoints. In contrast, intron numbers and length were conserved although all introns were not homologous between two genetically independent lineages and some isolates had a mix of introns from both lineages. Within the Tremellomycetes class, mitogenome length in 16 *Tremella fuciformis* isolates differed by 13,940 bp with both introns and intergenic regions contributing to the variation (Deng et al., 2020), while a smaller length difference (6,587 bp) was detected between 184 *Cryptococcus neoformans* mitogenomes mainly caused by variation in intron length (Wang and Xu, 2020).

The genus *Heterobasidion* (phylum Basidiomycota, class Agaricomycetes, order Russulales, family Bondarzewiaceae) consists of both saprotrophic and pathogenic species (Garbelotto and Gonthier, 2013). Species in the *Heterobasidion annosum sensu lato* (s.l) complex are all necrotrophic pathogens and cause root and heart rot in conifers in northern temperate regions (Woodward et al., 1998; Asiegbu et al., 2005). Five different species have been recognized in the complex: *H. irregulare* is found in the east and west of North America, and its hosts are *Pinus* spp., *Juniperus* spp., and incense cedar (*Calocedrus decurrens*). *Heterobasidion occidentale* is only found in western North America and has a broader host range of tree species in the genera *Abies, Picea, Tsuga, Pseudotsuga,* and *Sequoiadendron*. *Heterobasidion parviporum* is distributed from northern and central Europe all the way to southern Siberia where its main host, Norway spruce (*Picea abies*), is present. *Heterobasidion annosum sensu stricto* (s.s.) occurs in the whole of Europe except the most northern parts, further to the central parts of Asia and its main hosts are different *Pinus* species but it may infect Norway spruce. Finally, *H. abietinum* is located in central and southern Europe and its hosts are *Abies* species (Garbelotto and Gonthier, 2013). The species within the *H. annosum* s.l. complex are partially interfertile which suggests a potential for hybridization and possible exchange of mitochondria when the species exist in sympatry (Stenlid and Karlsson, 1991; Garbelotto et al., 1996, 2004).

The phylogeny of the complex suggests that their common ancestor first split into pine (*H. annosum* s.s. and *H. irregulare*) and non-pine (*H. parviporum*, *H. abietinum*, and *H. occidentale*) infecting species (Dalman et al., 2010). Although there is a pattern in the host preferences among the *H. annosum* s.l. species, the specialization has not been driven by co-speciation together with the host (Dalman et al., 2010). The common ancestor of *H. annosum* s.s./*H. irregulare* migrated from west Europe to eastern North America before the Atlantic land bridge disappeared and the two species *H. annosum* s.s. and *H. irregulare* evolved in allopatry for several millions of years in Europe and North America, respectively. The ancestor of *H. parviporum*, *H. abietinum,* and *H. occidentale* may have originated in east Asia or western North America (Dalman et al., 2010). The European species have a partly overlapping geographic distribution today. In North America, *H. irregulare* continued to spread west over the continent, while the spread of *H. occidentale* was restricted to the east by an arid region which probably was colonized by pines, which are not hosts for *H. occidentale* (Dalman et al., 2010). After the original speciation between pine and non-pine infecting species, *H. irregulare* and *H. occidentale* have had a long period to evolve in allopatry before today’s overlapping geographic distribution in western North America. How the evolutionary history with speciation in sympatry and a long period of allopatric evolution have shaped their nuclear and mitogenomes is not well understood.

It has been shown that the mitogenome content itself, rather than the whole mitochondrion, can have a significant role in fungal virulence (Zhan et al., 2004). Heterokaryotic hybrids created between fungal pathogen *Heterobasidion irregulare* and *H. occidentale* with nuclei from both parents but mitochondria from one of the parents showed virulence patterns similar to the parental isolate that contributed with its mitochondria to the hybrid (Olson and Stenlid, 2001).

The aims of this study were to (1) compare mitogenomes within the *H. annosum* s.l. complex in order to gain insight into the amount of variation in core genes, introns and intronic ORFs, intergenic regions, and its ORFs between the five species and within *H. parviporum, H. annosum,* and *H. abietinum* and (2) use the information of the variation to get a deeper understanding how mitogenomes evolve in fungi in general and in the *H. annosum* s.l. complex in particular.

## Materials and methods

### Sequencing and mitogenome assembly

In total, 60 isolates of *Heterobasidion* spp. were used in this study, 48 belonging to *H. parviporum*, seven to *H. abietinum*, three to *H. annosum* s.s., one to *H. occidentale,* and one to *H. irregulare* (Supplementary Table 1). The *H. parviporum*, *H. abietinum,* and *H. annosum* s.s. isolates originated from Europe and Ural region in Russia, while *H. occidentale* and *H. irregulare* originated in the USA.

DNA had previously been extracted from isolates of *H. parviporum*, *H. abietinum,* and *H. annosum* s.s. from a mycelium culture in liquid Hagem medium (20°C for 10 days) using the Qiagen Genomic tip columns 100 (Clergeot et al., 2018). A genomic library was prepared with an insert length of 400 bp as previously described (Dalman et al., 2013). Each library was sequenced from both ends with HiSeq 1,500 (Illumina, San Diego, CA) in order to generate paired-end reads of 150 bp. Quality control, trimming, *de novo* assembly of mitogenomes, and test for circularity were performed with NOVOPlasty (Dierckxsens et al., 2017). The mitochondrial gene *nad2* from *H. irregulare* isolate TC32-1 (Himmelstrand et al., 2014) was used as seed, and the TC32-1 mitogenome was used as a reference. All isolates were hence *de novo*-assembled in order to find larger rearrangements. The mitogenomes were edited so that all started at the same position before the ribosomal RNA (rRNA) gene *rnl*.

*Heterobasidion occidentale*, isolate TC122-12, where genome sequenced with PacBio RS II system (Pacific Biosciences, Menlo Park, CA) and assembled *de novo* at the Uppsala Genome Center (National Genomics Infrastructure in Uppsala, SciLifeLab, Sweden) using Hierarchical Genome Assembly Process version (HGAP3, Chin et al., 2013) corrected with Illumina reads clipped and filtered with Nesoni coming from the same isolate. A contig with the whole mitogenome was obtained by searching for mitochondrial genes with the blast tool. The *H. irregulare* mitogenome from strain TC32-1 and its annotation were obtained from previous studies (Olson et al., 2012; Himmelstrand et al., 2014).

### Annotation and comparative mitogenomics

All genes and ORFs in the *H. irregulare* TC32-1 mitogenome were aligned with blastx to the *H. parviporum* Sä159-5 and *H. annosum* Sä16-4 mitogenomes. Additional ORFs specific for the species were obtained in Artemis (Rutherford et al., 2000). A cutoff length of ORFs was set so that the probability of finding an ORF by chance was equal to finding a 420 bp ORF in *H. irregulare*, given the AT content of the species as done previously (Himmelstrand et al., 2014). Exon/intron boundaries of core genes were located by MUSCLE (Edgar, 2004) alignment with homologous genes from closely related species. DNA of ORFs and other intergenic and intron areas was analyzed with blastn and blastx to find similarities to genes from other species. The same procedure was done with the mitogenomes of *H. abietinum* and *H. occidentale* but using genes and ORFs from *H. parviporum*. The program tRNascan-se (Lowe and Eddy, 1997) was used to identify tRNA genes in all five species with settings “Sequence source”; Other mitochondrial and “Genetic Code for tRNA Isotype Prediction”: Mold & Protozoan Mito. Repetitions were located in the species with Rep-Seek (Achaz et al., 2007). All newly found intergenic ORFs were aligned against all five species of mitogenomes in order to find possible remnants of orthologue ORFs. The intergenic ORFs were translated to amino acids and used as tblastn queries in order to find homologous areas in other species. InterPro (Mitchell et al., 2019) and Phobius (Käll et al., 2004) were used to find domains, transmembrane topology, and signal peptides in the predicted amino acid sequences of the ORFs. The intergenic ORFs with unknown functions were named uORFs, ORFs with similarities to ORFs in other species located in mitochondrial plasmids were named putative plasmid ORFs (pplORFs), and regions with degenerated ORFs similar to intact hypothetical genes in *Heterobasidion* spp. or other species were named ORF relics (ORFrel).

In order to compare nucleotide distances in different mitogenome areas, four core genes (*atp9*, *cox1*, *nad2*, and *rps3*), three unknown ORFs (uORF6, uORF8, and uORF9), one relic ORF (uORF1rel), and three intergenic regions (one downstream of *cob*, one between *nad5* and *nad6*, and one between *rps3* and *nad2*) were aligned with Muscle (Edgar, 2004). The overall mean pairwise differences between the five species in the complex were calculated for all the genes, ORFs, and intergenic regions with the Kimura 2-parameter model, and dN/dS values were calculated for core genes and uORFs in MEGA version 7.0.14 (Kumar et al., 2016). MEGA version 7.0.14 was used to predict the best models for phylogenetic analysis that were used to construct phylogenetic trees of the 11 regions. The order of genes and intronic ORFs was extracted from Artemis files and was used to make an overview figure in order to analyze synteny and rearrangements between species. Total lengths of introns, core exons, rRNA, tRNA, and intergenic areas of the five *Heterobasidion* species and average lengths, differences in lengths, and differences in lengths/average lengths were calculated.

### Multiple sequence alignment and haplotype network

A multiple sequence alignment (MSA) of 48 *H. parviporum* mitogenomes was performed with MAFFT (Katoh et al., 2002; Katoh and Standley, 2013). There were two very different forms of introns in *cox 1* (intron 10a and 10b) that had to be aligned separately. Intron 10a and 10b in *cox1* and the containing intronic ORFs were used as queries for blastn and tblastn searches against the nr database at the National Center for Biotechnology Information (NCBI) and the mitogenomes of Sä16-4 and TC32-1. Intronic ORFs 10a and 10b in *cox1* were analyzed with InterPro (Mitchell et al., 2019), and SignalP-5.0. A Python script was created to filter out single-nucleotide polymorphisms (SNPs) and indels from the MSA. Intron 10 in *cox1* was removed, and a NEXUS file was produced in AliView (Larsson, 2014) for further analysis of network structure in Popart (Leigh and Bryant, 2015). Ten *H. parviporum* isolates were analyzed with MToolBox (Calabrese et al., 2014) to identify signs of heteroplasmy. The program was adapted to the high AT content and the abundance of homopolymer regions.

## Results

### Mitogenome content of *Heterobasidion annosum* s.l. species

**Figure 1:**
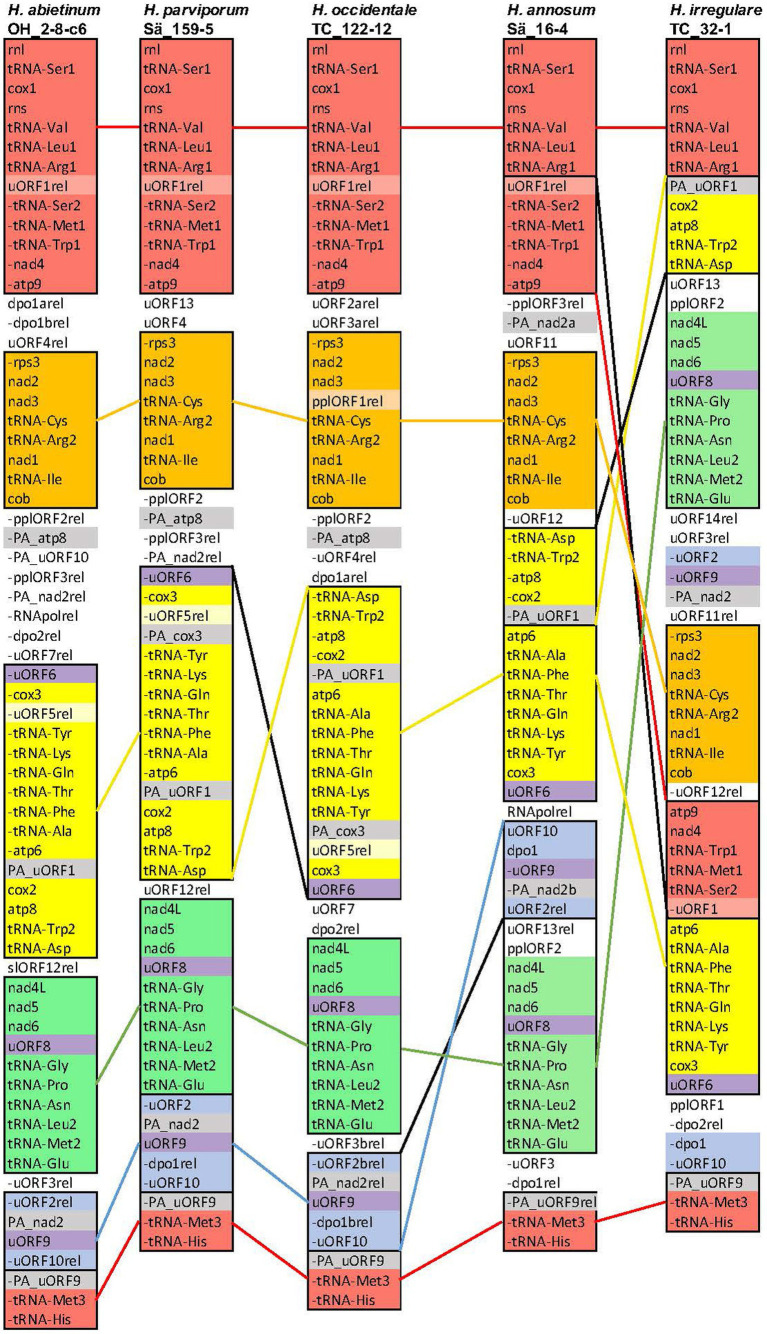
Synteny and rearrangements of the mitogenomes of the five *H. annosum* s.l. species. The organization of genes and intergenic ORFs of the mitogenomes of one representative isolate from each of the five *H. annosum* s.l. species. Colored blocks represent conserved synteny areas. The three uORFs that are (Continued)FIGURE 1 (Continued)present in all five species are colored purple. A minus sign (−) before the name means that the gene/ORF is located on the opposite strand. Colored single lines between species represent conservation or translocation and, in combination with a black line, represent inversions.

The complete mitogenomes of 60 isolates of five *Heterobasidion annosum* s.l. species were sequenced and *de novo*-assembled. The mitogenome lengths varied with a maximum difference of 7,298 bp between *H. abietinum* (116,176 bp, Faf6_3) and *H. annosum* s.s. (108,878 bp, Sa16_4) (Table 1). All five species shared the same 15 core genes (*nad1*, *nad2*, *nad3*, *nad4*, *nad4L*, *nad5*, *nad6*, *cob*, *cox1*, *cox2*, *cox3*, *atp6*, *atp8*, *atp9*, and *rps3*), two rRNA, and 26 tRNA. In contrast to the similarities in overall length and core gene present, there were variations in the number of introns, intronic ORFs, and intergenic ORFs (Table 1). The largest variation in lengths was found in the introns whereas the difference in intergenic areas was intermediate and the genetic regions were the lowest (Supplementary Table 2). The 5′ ends of three partial core genes (PA_nad2, PA_cox3, and PA_atp8) and two partial uORFs (PA_uORF1 and PA_uORF9) were located upstream of *cox2* and of intergenic ORFs (Figure 1). In the multiple alignments of the five species in the intergenic area between *nad5* and *nad6,* a 13 bp long GC-containing self-complementary palindrome (CCGCCGCCGGCGG) was found in seven places and they were optional in the presence/absence in the five *Heterobasidion* species (Supplementary Figures 1a and 1b). Blastn with the motif as query and *H. annosum* Sä159-5 mitogenome as search set showed that the motifs were located in introns and intergenic regions, but they were only located in between or beside the core genes and RNA genes and not in the regions with only intergenic ORFs. One GC motif was in an intronic ORF relic in intron five of *cox1*. Another was in the frame in the last bases in the 3′ end of an intact intronic ORF of intron four of cob. The full 13 bp sequence of the GC palindrome was found in 52 places in *H. irregulare* TC32-1, 49 in *H. parviporum* Sä159-5, 44 in *H. abietinum* OH_2-8-c6, and 30 each in *H. occidentale* TC_122–12 and *H. annosum* Sä_16-4 (Supplementary Table 3). We did not find these GC palindromes in any other mitogenomes in the order of Russulales.

**Table 1:** Mitogenome content of *Heterobasidion annosum* s.l. species.

	*H. ann* s.s.	*H. irr*	*H. par*	*H. abi*	*H. occ*
Genome length (bp)	108,878–108,904	114,193	112,367–112,709	113,143–116,176	113,820
Introns (in total)	25	26	28	29	29
*cob*	7	7	7	7	7
*cox1*	10	10	10	10	9
*cox2*	2	2	3	3	3
*cox3*	2	2	2	2	2
*nad1*	2	2	2	2	2
*nad5*	1	1	1	1	1
*rnl*	1	2	3	4	5
HEGs	11	12	14	14/15*	11
HEG relics	3	4	3	4	5
Intergenic ORFs	7	6	7	4	4
Intergenic ORF relics	3	5	3	7	6
Plasmid ORF	2	2	1	0	1
Plasmid ORF relics	3	2	2	5	3

* Different number of intronic ORFs in different isolates of *H. abietinum*. H. ann, *H. annosum* s.s.; H. irr, *H. irregulare*; H. par, *H. parviporum*; H. abi, *H. abietinum*; H. occ, *H. occidentale*.

### Synteny between species

The synteny and rearrangement of the mitogenome of the five *H. annosum* s.l. species were analyzed and compared with the phylogenetic species. There was an overall synteny of conserved blocks of the core genes between the species (Figure 1). The mitogenome structure of three non-pine infecting species *H. parviporum, H. abietinum,* and *H. occidentale* was fairly similar. There was one major rearrangement between *H. occidentale* and *H. parviporum/H. abetinum,* a large inversion that included the core genes *atp8*, *cox2*, *atp6,* and *cox3*, seven tRNA genes, and some intergenic ORFs (Figure 1, yellow block). However, in two intergenic areas between *atp9* and *rps3* and between *cob* and uORF6, there was a substantial extent of rearrangements of intergenic ORFs. Between the non-pine infecting group of species and *H. annosum* s.s., one translocation and inversion of an area with intergenic ORFs have taken place (Figure 1, blue block). The organization of the mitogenome of *H. irregulare* differed substantially from the other species, and the high number of genomic rearrangements (3 or 4 translocations and 2 or 3 inversions) did not correspond to its phylogenetic distance to the other species. Without exceptions, the rearrangement breakpoints were located in areas with intergenic ORFs or partial ORFs. Most of the intergenic ORFs were located in separate areas apart from the core genes, and there were a massive number of rearrangements among them. However, two intergenic ORFs were located in the same areas in all five species: The uORF8 within a block (green) of core genes and uORF6 located at the end of another block (yellow) of core genes. There was also one block (blue) of intergenic ORFs that had a more conserved synteny containing the uORF9 flanked with a duplicated part of nad2 (PA_nad2).

### Open-reading frames in intergenic regions of *H. annosum* s.l. mitochondria

**Figure 2:**
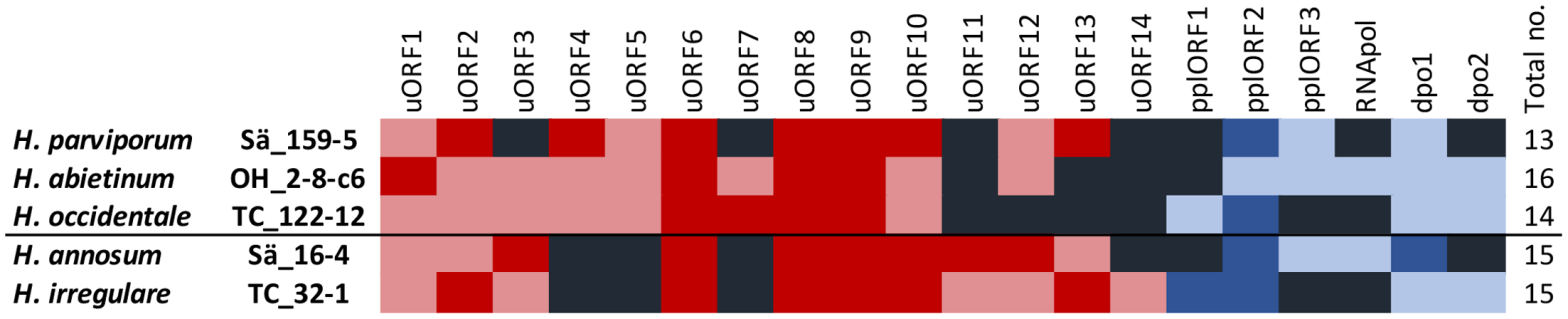
Intergenic ORFs of the mitogenomes of the five *H. annosum* s.l. species. Colored boxes represent intact uORFs (red), uORF relics (light red), lack of ORF (black), intact plasmid ORF (blue), and plasmid ORF relics (light blue). The black line divides the species into pine and non-pine infecting ones.

In the five *H. annosum* s.l. species, 20 different intergenic ORFs and sequences with similarity to ORFs but with frame shifts and premature stop codons, referred to as ORF relics (ORFrel), were found. The number varied between 13 and 16 in the different species (Table 1; Figure 2). Out of these, 14 had significant homology to mitochondrial ORFs or mitochondrial plasmid ORFs of other species while six did not have homology with any known genes in the GenBank database (Supplementary Table 4). The ORFs with no similarities to mitochondrial plasmid ORFs and with unknown functions are referred to as uORFs. Three of the uORFs were present and intact in all five species (uORF6, uORF8, and uORF9). In one or more of the five *Heterobasidion* species, 17 of the intergenic ORFs contained frame shifts and premature stop codons, but still with similarities (E-value ≤1e-20) to intact ORFs found in one or more of the *H. annosum* s.l. species or to mitochondrial ORFs in other species (Supplementary Table 4). Six ORFs and ORF relics were associated with mitochondrial plasmids (Table 1; Supplementary Table 4). One intact ORF (*dpo1*) in Sä16-4 had high sequence similarity to a DNA-directed DNA polymerase family B, and in addition, there was one other *dpo* relic variant (*dpo2rel*) found. The *dpo1* is present as relics in the other four *H. annosum* s.l. species, while *dpo2rel* is present in *H. abietinum*, *H. occidentale,* and *H. irregulare*. One relic ORF (RNApolrel) in *H abietinum,* isolate OH_2_8_c6, was similar to a DNA-directed RNA polymerase in *Tricholomella constricta,* and there was also a more degenerated relic of this gene in *H. annosum s.s.*, isolate Sä16-4. Three other ORFs (pplORF1, pplORF2, and pplORF3rel) had similarities to mitochondrial plasmid genes from other fungal species (Supplementary Table 4). Analysis of the predicted protein sequences of the 20 intergenic ORFs by InterProScan showed that nearly all had transmembrane regions except for DPO1, RNApolrel, and uORF7 (Supplementary Table 4). Two of the putative plasmid ORFs (pplORF2 and pplORF3rel) had signal peptides predicted by Phobius. It is only uORF14 that is unique to *H. irrgeulare* (Figure 2) although it has sequence similarities to ORFs in other Agaromycete species. The distribution of the intergenic ORFs among species correlates with the two phylogenetic groups, pine infecting/non-pine infecting *Heterobasidion* species for uORF4, uORF5, and uORF11 but not for the remaining uORFs.

### Nucleotide distances in shared ORFs of *H. annosum* s.l. species

The nucleotide distances were analyzed for three uORFs (uORF6, uORF8, and uORF9) that were present in all five *Heterobasidion* species. These ORFs were intact and located in areas with conserved synteny (Figure 1) and were compared with intergenic regions, uORF1rel, and core genes. The nucleotide distances for uORF6 and uORF9 were similar to the values for intergenic regions, while uORF8 had a value comparable to the core genes (Table 2). Both uORF6 and uORF9 had notably high dN/dS values of 1.15 and 0.9, respectively, while uORF8 had a value close to that of core genes. The degenerated uORF1rel with stop codons and frameshifts was also present in all five *Heterobasidion* species, but the nucleotide distances were higher than for the intergenic regions and intact uORFs. Phylogenetic trees with the uORFs and intergenic regions were all congruent with the evolutionary history of the *Heterobasidion* species.

**Table 2:** Mean nucleotide distances for conserved ORFs, core genes, and intergenic regions of *H. annosum* s.l.

	Nucleotide distance^a^	dN^b^	dS^c^	dN/dS^d^
uORF6	0.038	0.038	0.033	1.15
uORF8	0.011	0.008	0.023	0.35
uORF9	0.046	0.046	0.051	0.90
*atp9*	0	0	0	n.a.
*cox1*	0.012	0	0.049	n.a.
*nad2*	0.015	0.011	0.029	0.38
*rps3*	0.014	0.010	0.029	0.34
IGR cob downstr	0.058	n.a.	n.a.	n.a.
IGR nad5-nad6	0.048	n.a.	n.a.	n.a.
IGR rps3-nad2	0.035	n.a.	n.a.	n.a.
uORF1rel	0.071	0.063	0.097	0.65

^a^Average nucleotide distance calculated using the Kimura 2-parameter model, ^b^non-synonymous substitution per non-synonymous sites, ^c^synonymous substitution per synonymous sites, and ^d^estimate of selection.

### Introns and intronic ORF content of *H. annosum* s.l. species

**Figure 3:**
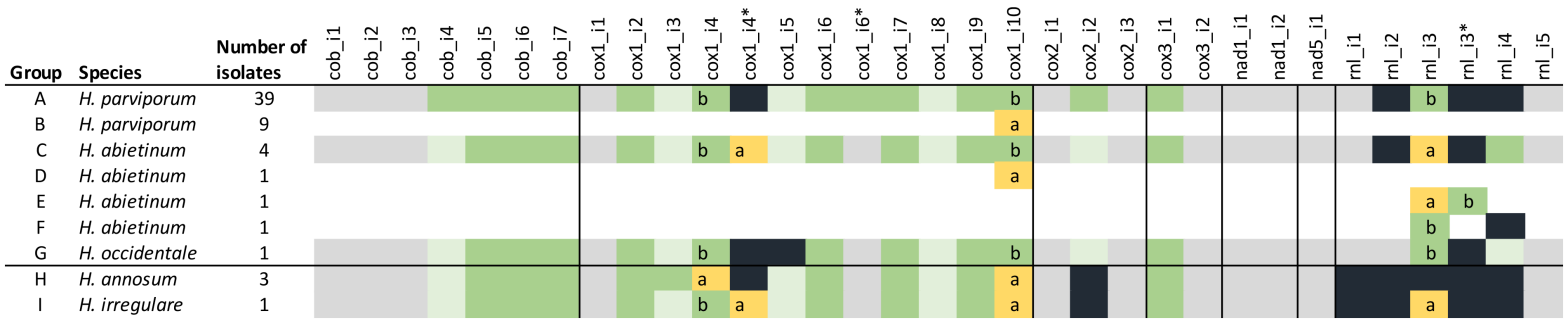
Introns and intronic ORFs of the mitogenomes of 60 isolates of the five *H. annosum* s.l. species. Colored boxes represent intron (gray), intron with intact HEG (green), intron with intact HEG variant (yellow), intron with HEG relic (light green), and no intron/HEG (black). The black line divides the species into pine and non-pine infecting ones. Letters a and b represent the two different variants of introns, and * indicate an additional HEGs position in an intron. Isolates included in the different groups are found in Supplementary Table 1.

Overall, the number and locations of introns were well conserved in most genes of the species. Six core genes (*cox1*, *cox2*, *cox3*, *cob*, *nad1*, and *nad5*) and *rnl* had introns and five of them had homing endonuclease genes (HEGs) in some introns (Figure 3). In total, there were 30 intron locations, and there were 20 locations with HEGs or HEG relics in 17 introns. The number of introns varied between 25 and 29 and intact HEGs between 11 and 14 between species (Figure 3). All five species shared 24 of the 30 intron locations, but only eight of the 20 HEGs were intact and conserved in all five species. In 14 introns, the HEGs were homologous in the same introns although with varying levels of sequence similarity. Three introns, namely cox1_i4, cox1_i10, and rnl_i3, had two alternative forms of HEGs in the different species, and in cox1_i4, both HEG forms were present in both *H. abietinum* (OH_2-8-c6) and *H. irregulare* (TC_32-1). *Cox1* had the largest amount of ten introns in four of the *Heterobasidion* species although *H. occidentale*, TC122-12 had only nine introns. A striking exception from the conservation pattern was in the gene *rnl* where the number of introns varied between one and five in the *H. annosums* s.l. species complex. The only correlated patterns with the two phylogenetic groups pine infecting/non-pine infecting *Heterobasidion* were with one extra intron in *cox2* and *rnl* and two different kinds of HEGs in intron 10 of *cox1* (io-cox1 10a and 10b). Although some isolates of *H. parviporum* and *H. abietinum* also had the cox1 10a intron form, the io-cox1_i10a had E-values down to 4,00E-130 (*Auricularia polytricha*) and similarities up to 69% (*Fomitiporia mediterranea*) to other species mitogenomes. In comparison, io-cox1_i10b had higher E-values (down to 2,00E-41 in *Porodaedalea pini* strain BCRC 35384) and lower similarities (up to 45% in *Smittium culisetae*) to the same positions in the same species as io-cox1_i10a had similarities too. The two different intronic ORFs in cox1_i10 (io-cox1_i10a and io-cox1_i10b) both belong to the GIY-YIG endonuclease superfamily and contained a DNA-binding domain of the intron-encoded endonuclease superfamily.

### Diversity of *H. parviporum* mitogenomes

Among the mitogenomes of 48 *H. parviporum* isolates, no rearrangements were found and the overall SNP frequency of all isolates was 0.28% (317 SNPs) and indel frequency 0.043% (48 indels). The SNP frequency of the core genes was only 0.09% and ten of them had no SNPs, whereas *nad2* and *rps3* had four, *cox1* and *nad5* had two, and *nad3* had one SNP. Twenty 1–4 bp long indels were positioned at homopolymeric areas. The longest indels (5–30 bp) were in six tandem repeat areas. None of the 15 core genes had indels, and only one of the intergenic ORFs (uORF9) in isolate (87_215_3) had an in-frame indel. No evidence of heteroplasmy was found within any of the isolates.

There were two different lengths of the mitogenomes found among the 48 *H. parviporum* isolates. Nine of the isolates had a shorter length of 112,367–112,396 bp, whereas the other 39 had lengths between 112,654 and 112,709 bp. The differences in lengths between the two groups were due to the exchange of intron 10 in *cox1* (io-cox1_i10a and io-cox1_i10b) (Figure 3). There was a high similarity between intron cox1_i10a in *H. parviporum* and the same cox1 intron location in *H. annosum* (98.97%) and *H. irregulare,* (98.10%).

**Figure 4:**
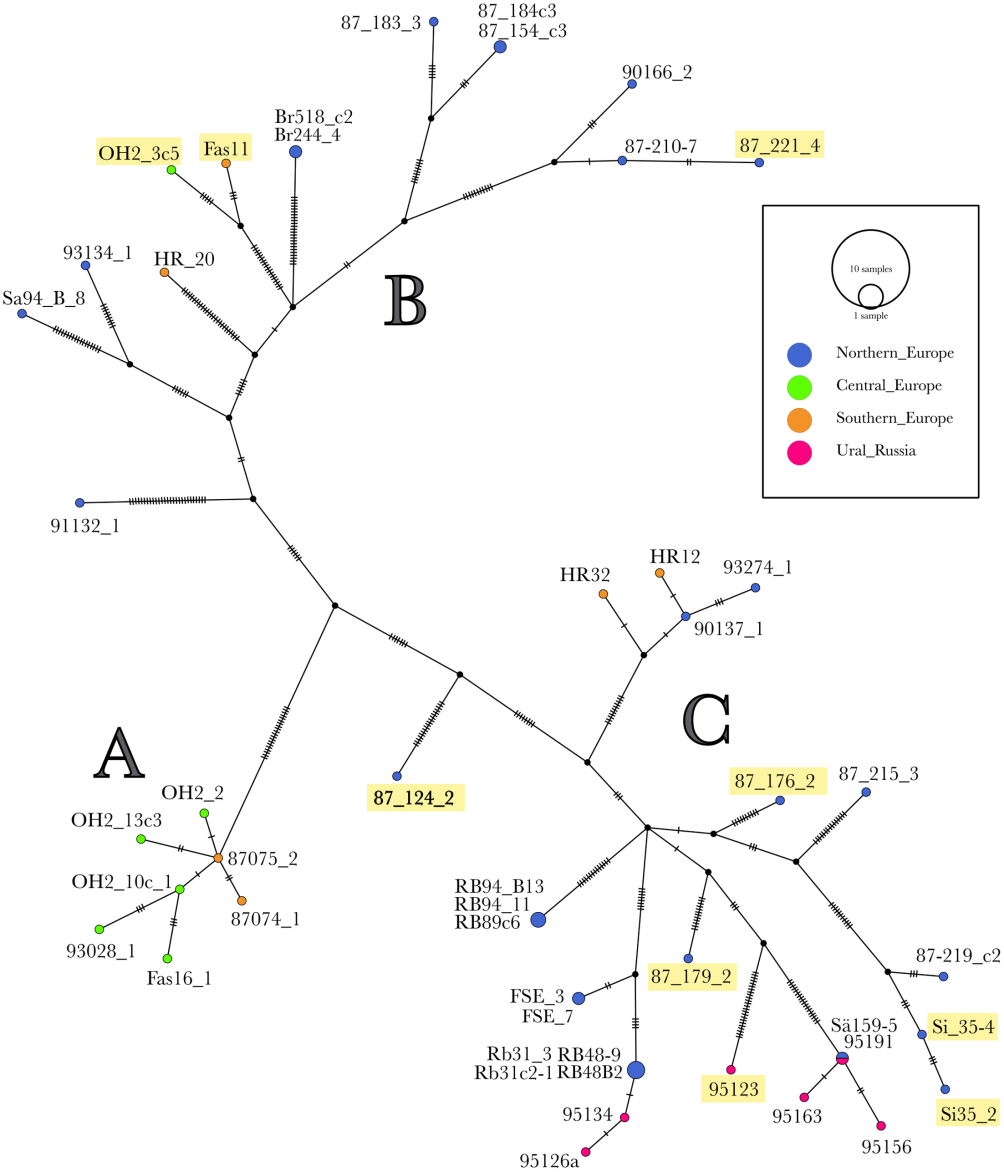
Haplotype network of the 48 *H. parviporum* isolates. A haploid network of the SNP found in the mitogenomes of 48 *H. parviporum* isolates created using Popart. Each vertical tick represents one mutation difference. Isolate names underlined with yellow contain the intron variant cox1_i10a.

The haplotype network of the 48 *H. parviporum* isolates whole mitogenomes constructed by Popart showed three groups: A, B, and C (Figure 4). Although there is not a full congruence with geographic origin, some patterns can be found. Group A consists of seven isolates from central and southern Europe with low variation between them. All isolates from Ural_Russia form one group C together with some isolates from northern Europe and two from southern Europe. Group B consists of northern European isolates but also a few from central and southern Europe. The nine isolates with the alternate intron 10a in cox1 were spread out in six different places in groups B and C, but four of these isolates were sampled in Oslo, Norway, and two in Siarö, Sweden.

### Diversity of *H. abetinum* and *H. annosum* mitogenomes

The organization and content of the mitogenomes of the seven analyzed *H. abietinum* isolates were very similar. Four of the seven *H. abietinum* mitogenomes had very similar lengths between 114,829 and 114,849 bp. Isolate Faf6_3 was longer (116,176 bp) due to a 1,352 bp extra part of intron rnl_i3 (same intron and HEG as in *H. parviporum*, Sä_159–5 and *H. occidentale*, TC_122-12) (Figure 3). Isolate OH_2-9_c-3 was shorter (113,143 bp) as a result of an alternative intron rnl_i3 and the lack of intron rnl_i4. Isolate OH2_14c_4 had the same intron exchange in intron 10 of cox1 as was seen in the population of *H. parviporum* isolates. Sequence identity between intron cox1_i10a of *H. abietinum* OH2_14c_4 and *H. parviporum* 87_124_2 was 99.6%. Lengths of the three *H. annosum* isolates differed with only 26 bp (108,878–108,904 bp). None of them had intron variants or rearrangements.

## Discussion

Compared with other genera in Russulales and other Agaricomycetes, mitogenome variation between the five *H. annosum* s.l. species and isolates within *H. parviporum* is low. The variation of lengths is small and so are the nucleotide distances. Introns, HEGs, and intergenic ORFs display a high degree of homology between the five *Heterobasidion* species. In the 48 *H. parviporum* mitogenomes, there was one single intron difference, and SNP frequencies were low. Mitogenome length differences in the *Heterobasidion* genus are considerably smaller compared to the *Russula* and *Lactarius* genera from the same order (5,951 bp vs. 22,398 and 28,462 bp, respectively) (Li et al., 2018, 2019a). In other studied basidiomycetes, the mitogenome length differences within genera have been between 1,952 bp in two *Coniophora* species (Wu et al., 2021) and 117,845 bp in two *Pyrrhoderma* species (Lee et al., 2019).

The positions and synteny of core genes and intergenic ORFs in *H. annosum* s.l. are considerably less conserved than in *Russula* and *Lactarius*. The six *Lactarius* species have not any rearrangements of core genes, and in the six *Russula* species, only atp6 has an altered location in one of the species (Li et al., 2018, 2019a). Taken together, these data suggest a lower substitution frequency and more frequently occurring mitogenome rearrangements in *Heterobasidion*, compared with *Russula* and *Lactarius.*

The unknown intergenic ORFs of fungal mitogenomes are often species-specific, but some are shared within genera as, for example, in four closely related *Rhynchosporium* species (Torriani et al., 2014). Thus, these ORFs could be involved in activities that are specific to the species or genera. However, there are not many detailed studies made on these unknown intergenic ORFs. As many intergenic ORFs are shared among several of the *H. annosum* s.l. species, this genus may serve as a good system to study the origin, evolution, and function of intergenic ORFs. Most intergenic ORFs and relics were present in species of both the non-pine and the pine-infecting phylogenetic groups. This suggests that these were present before the divergence of the groups. A difference between the uORFs and the putative plasmid ORFs was that the uORFs only had similarities to other Agaricomycetes mitogenomes whereas three out of six putative plasmid ORFs had similarities to Ascomycetes (Supplementary Table 4). This is in agreement with a study in Agaricomycetes where unknown ORFs were only shared between closely related species (Araújo et al., 2021) and another phylogenetic study of *dpo* and *rpo* homologs where some clades had species from different phyla (Fonseca et al., 2021). In the five *Heterobasidion* species, *dpo1* had even similarities to mitochondrial ORFs in the plant class of Magnoliopsida which could be a sign of a horizontal transfer event. Evidence was earlier found that species in the phylum of Glomeromycota could be donors of mitochondrial plasmids to plants (Beaudet et al., 2013).

Three uORFs, uORF6, uORF8, and uORF9, were particularly interesting since they were intact in all five *Heterobasidion* species and were located in areas with conserved synteny. The fact that uORF8 had low K2P distances and dN/dS values comparable with the core genes (Table 2), which should be under purifying selection, suggests that uORF8 could have a conserved function for all the *H. annosum* s.l. species. This agrees with another study in *H. parviporum* where uORF8 (previously called nc-ORF5), together with two mitochondrial proteins encoded in the nuclear genome, is involved in radial growth rate (Clergeot and Olson, 2021). Since the uORF8 only had similarities to other ORFs of species in the order Russulales, it appears to be specific to this order (Supplementary Table 4). At present, the functionalities of uORF6 and uORF9 are more uncertain since they are very variable at both nucleotide and amino acid levels with dN/dS values close to one. Interestingly, uORF6 has no similarities to ORFs in any other species in public databases than the five *Heterobasidion* species (Supplementary Table 4). In a comparative study of the nuclear genomes of *H. irregulare* and *H. annosum s.s.,* the majority of genes under positive selection were involved either in transcriptional or mitochondrial functions (Sillo et al., 2015). In view of this and our prior knowledge that the mitogenome of hybrid *Heterobasidion* species controls their virulence (Olson and Stenlid, 2001), the uORF6, uORF8, and uORF9 could be suitable candidates for further evaluation.

The observation that the 5′ end partial genes and ORFs are frequently present in putative promoter regions of ORFs in Heterobasidion mitogenomes suggests that gene duplication events may drive diverging transcriptional regulatory patterns. It is known that the first codons in the 5′ end often have signals important for the initiation and the elongation steps of translation and consequently are important for gene regulation (Tuller and Zur, 2015). Duplications of core genes have been observed in other species (Paquin et al., 1994; Al-Reedy et al., 2012; Kanzi et al., 2016; Deng et al., 2020). The duplications may derive from partial cDNA copies of mitochondrial transcripts as mitogenomes often contain mobile group II introns with reverse transcriptase-encoding elements (Al-Reedy et al., 2012).

The GC palindromes found in the *H. annosum* s.l. complex resemble the GC clusters (or byp-like sequences) in *Saccharomyces* species mitogenomes that are well known. The GC clusters are dispersed mostly in the otherwise very AT-rich intergenic regions, are between 30 and80 bp long, are palindromic with hairpin secondary structures, and have a relatively high GC content (de Zamaroczy and Bernardi, 1986; Weiller et al., 1989). The existence and locations of these GC clusters are optional in the presence/absence of both between and within *Saccharomyces* species (Wolters et al., 2015; Sulo et al., 2017) and horizontal transfer of them has been documented (Lang et al., 2014; Wu and Hao, 2015). GC-clusters contains putative target-site duplications and are proposed to be transposable elements that proliferate through RNA-mediated retrotransposition facilitated by reverse transcriptases encoded in intronic ORFs (Séraphin et al., 1987; Weiller et al., 1989; Lang et al., 2014; Wu and Hao, 2015). The GC palindromes are variable in number and location in the five *Heterobasidion* species and could be a transposable element that appears to be specific for the *Heterobasidion* genus. Since there was no variation in the locations of GC palindromes within the 48 *H. parviporum* isolates, the GC palindromes are probably not currently active in this species.

Similar short optional repeats have been found in other fungal and plant species (Yin et al., 1981; Nargang et al., 1984; Gross et al., 1989; Boer and Gray, 1991; Koll et al., 1996; Paquin and Franz Lang, 1996; Paquin et al., 2000; Jiao et al., 2004; Formey et al., 2012; Beaudet et al., 2013; Deng et al., 2018; Liu et al., 2020; Ponts et al., 2020) in the fungal phyla of *Ascomycota, Chytridiomycota, Mucoromycota,* and *Blastocladiomycota* and recently in *Basidiomycota*. A connection between rearrangements and the short optional repeats has been seen in a number of studies where repeats were more abundant in genera with many rearrangements, and they were often located in rearrangement breakpoints (Koll et al., 1996; Nedelcu and Lee, 1998; Bullerwell et al., 2003; Kim et al., 2018; Zhang et al., 2019). However, it has also been argued that short optional repeats could have a functional role, for example, in affecting the supercoiling of the DNA, in the position of nucleosomes, in the formation of other secondary structures of DNA, in directly interacting with proteins, by hindering degradation of the mRNA, and as regulatory elements (de Zamaroczy and Bernardi, 1986; Pearson et al., 1996; Jiao et al., 2004). André et al. (1992) proposed that some small repeats could be derived from the reverse transcription of non-functional parts of mRNA. Mutants of *Neurospora crassa* had plasmid-like elements in their mitochondria with tandem repeated 125–296 bp monomers (Hausner et al., 2006). Each of these monomers contained a GC-rich palindrome, a promoter, and the 5′ terminal region of the rRNA gene rnl. The authors of this study suggested that the GC-rich palindromes could be replication fork pausing points in a recombination-dependent rolling-circle replication system. This could suggest that the same reverse transcription mechanism is involved in the formation of duplications of partial genes as in the spread of short palindromic repeats.

Variation in the presence/absence of introns and intronic ORFs occurs frequently in mitogenomes within fungal genera, and it is one of the most common reasons for length variations (Li et al., 2018, 2019a, 2022a; Lee et al., 2019). The five *Heterobasidion* species in our study stand out in intron variation considering that there are only minor differences between them. Although there were 30 intron locations in total, only six intron locations varied in presence/absence. The other 24 introns were fixed in all five species and were most likely present before the division of the five species. More recent activity after the divergence seems to have occurred in the rRNA gene rnl where four out of the five introns varied. The intronic HEGs are also conserved in sequence similarity and locations in comparison with other genera. In 17 introns with HEGs, only three introns had two alternative forms of HEGs in the different species. Most of the movement of the HEGs, therefore, appears to have happened before the divergence of the five species. In all, three intron locations (cox1_i10, cox2_i2, and rnl_i1) had differences between but not within the pine and non-pine infecting groups. These differences might have arisen after the divergence of the pine and non-pine infecting groups but before the divergence of the five species of today. Six intron locations out of 30 (cox1_i4, cox1_i5, cox1_i6, rnl_i2, rnl_i3, and rnl_i4) had differences that probably occurred after the divergence of all five species since they appeared sporadically in the two groups. There was also some intron variation within the species *H. parviporum* in intron cox1_i10 and *H. abietinum* in introns cox1_i10, rnl_i3, and rnl_i4. Hence, intron variation seems to have occurred mostly before, but also during and after the divergence of the five *Heterobasidion* species. The variation in intron lengths is due to the presence/absence of introns and HEGs, probably as a result of horizontal gene transfer (Burt and Koufopanou, 2004; Stoddard, 2005).

In comparison with other within-species studies of mitogenomes, the 48 *H. parviporum* isolates had a short length difference of only 342 bp between the longest and shortest mitogenomes. In other within-species studies of basidiomycetes, the length differences were 6,587 bp in 184 *Cryptococcus neoformans* isolates, 13,940 bp in 16 *Tremella fuciformis* isolates, and 22,174 bp in 59 *Phellinus noxius/Pyrrhoderma noxium* isolates (Lee et al., 2019; Wang and Xu, 2020). Length variations were caused by both intron and intergenic regions in *Phellinus noxius/Pyrrhoderma noxium* and *Tremella fuciformis* but only by introns in *Cryptococcus neoformans*. In fact, in all these three Basidiomycete within-species studies, the mitogenome length differences are even larger than between the five *Heterobasidion* species in this study that differs in length with 5,951 bp. The core gene SNP frequency of 0.09% in the 48 *H. annosum* isolates was among the lowest in comparison with other studies although not many numbers are reported. A study of 9 *de novo*-assembled and 41 reference-assembled *Lachancea thermotolerans* isolate mitogenomes showed a core gene SNP frequency of 0.77% (Freel et al., 2014), whereas in 24 *de novo-*assembled *Fusarium graminearum* isolates it was only 0.02% (Brankovics et al., 2018).

Presence/absence variation of introns in core genes is common within species (Wolters et al., 2015; Brankovics et al., 2020; Deng et al., 2020; Kulik et al., 2020; Wang and Xu, 2020; Yang et al., 2020). It is rare with no presence/absence intron variation at all as within the 48 *H. parviporum* isolates. However, there was an exchange of two different kinds of introns in intron cox1_i10 where 9 isolates had one kind of intron, cox1_i10a, and the other 39 isolates had another kind, cox1_i10b. These kinds of exchanges of introns are not as well studied as presence/absence intron variation. But in the study of 59 *Phellinus noxius/Pyrrhoderma noxium* mitogenomes, an extensive exchange of introns between two lineages was revealed where some strains harbored a mosaic origin of introns from both lineages (Lee et al., 2019). It was proposed that the chimeric pattern was a result of recombination. The most common explanation for intron movement is otherwise horizontal gene transfer (Burt and Koufopanou, 2004; Stoddard, 2005). Several of our nine isolates with the alternative intron were sampled in the same regions. This would have made it possible to transfer the alternative intron between the isolates.

The part correlation between the haplotype group and the geographic location in the haplotype network of the whole mitogenomes of the 48 *H. parviporum* isolates could be due to that most of the effective spore dispersal in Heterobasidion is local (Stenlid, 1994), but the occasional long-distance dispersal, perhaps enhanced by human activity, may help to contribute to gene flow. The fact that isolates from the same geographic regions were represented in different phylogenetic clades in 184 mitogenomes of *Cryptococcus neoformans* was proposed to be explained by its ability of long-distance dispersal and a variety of ecological niches (Wang and Xu, 2020).

The mitochondria of OH2_10c_1 had a 100% query cover and 99.9% identity with the *H. parviporum* isolate Sä159-5. However, the isolate nuclear genome has been classified as belonging to *H. abietinum* (Unpublished data). This indicates that the isolate could be a result of hybridization between the two species. Different *Heterobasidion* species are known to be compatible in the laboratory (Olson and Stenlid, 2001; Oliva et al., 2011), and there is field evidence of hybridization between *H. irregulare* and *H. occidentale* (Garbelotto et al., 1996, 2004) and between *H. irregulare* and *H. annosum* (Sillo et al., 2019).

Two characteristic features of the five species of *Heterobasidion* mitogenomes are the findings of partially duplicated genes/ORFs and the GC palindromes. Since all rearrangement breakpoints are in areas with intergenic ORFs and partial genes/ORFs, it can be assumed that something in these areas is important to the mechanism causing the rearrangements. If the partially duplicated genes/ORFs are involved in the expression of the immediately downstream genes and ORFs, they could also be crucial factors to ensure that all vital genes are continuing to be expressed after rearrangements have occurred. The GC palindromes are only located in the vicinity of core or rna genes and are therefore probably not directly involved in the mechanism for rearrangements. They might instead have a stabilizing role for the blocks of core genes as was suggested in Cahan and Kennell (2005). This could enable frequent reshuffling without disturbing the important functions of the core genes. The partially duplicated genes/ORFs may also be important factors to maintain the expression of integrated intergenic ORFs. This could explain how the uORF9, that is, downstream of the partial *nad2* gene in a block with intergenic ORFs, has not degenerated and could be functioning in all five *Heterobasidion* species. The two other intergenic ORFs, uORF6 and uORF8 that are intact in all *H. annosum* s.l. species, are integrated into blocks with core genes and might be expressed with the help of regulating elements of the core genes. The maintaining of the expression of uORF6, uORF8, and uORF9 could then have made it possible to evolve into new functions specific to their species, but this remains to be further investigated in future studies.

This study has given new insights into the mitogenome variation at the genus and within the species level. Despite relatively low sequence variation between the five *Heterobasidion* species, they have, in comparison with other genera, a surprisingly high frequency of rearrangements of intergenic ORFs and core genes. This could be a key clue for the understanding of the mitochondrial evolution of *H. annosum* s.l. The role of the partially duplicated genes/ORFs and the GC palindromes in making the five *Heterobasidion* mitogenomes more susceptible to rearrangements is not known at the moment. This would be an interesting question to address in future studies.

## Data availability statement

The original contributions presented in the study are publicly available. This data can be found here: Genbank, accession numbers OQ842904-OQ842961.

## Author contributions

KH and MB analyzed the data. KH and ÅO wrote the manuscript. All authors contributed to the design of the experiments, and read, reviewed, and approved the final manuscript.

## Funding

The authors would like to acknowledge support of the National Genomics Infrastructure (NGI)/Uppsala Genome Center and UPPMAX for providing assistance in massive parallel sequencing and computational infrastructure. Work performed at NGI/Uppsala Genome Center has been funded by RFI/VR and Science for Life Laboratory, Sweden. The Swedish Research Council for Environment, Agricultural Sciences and Spatial Planning (FORMAS) Biomassa grant 942-2025-81 to JS is acknowledge.

## Conflict of interest

The authors declare that the research was conducted in the absence of any commercial or financial relationships that could be construed as a potential conflict of interest.

## Publisher’s note

All claims expressed in this article are solely those of the authors and do not necessarily represent those of their affiliated organizations, or those of the publisher, the editors and the reviewers. Any product that may be evaluated in this article, or claim that may be made by its manufacturer, is not guaranteed or endorsed by the publisher.

## Footnotes

^1^ https://www.ncbi.nlm.nih.gov/genome/browse#!/organelles/

^2^ https://www.ncbi.nlm.nih.gov/nuccore/NC_056148.1
