# Supplementary material for: Multiple rearrangements and low inter- and intra-species mitogenome sequence variation in the Heterobasidion annosum s.l. species complex
Source: Front Microbiol. 2023 May 18;14:1159811. doi: 10.3389/fmicb.2023.1159811 (PMC10234125; doi:10.3389/fmicb.2023.1159811)
Supplement: Supplementary file 2 [file Data_Sheet_1.PDF]

Supplementary Figure 1a: GC palindrome

```
CCGCCG\
||||||  C
GGCGGC/
```

Supplementary figure 1b: Indels with GC palindromes

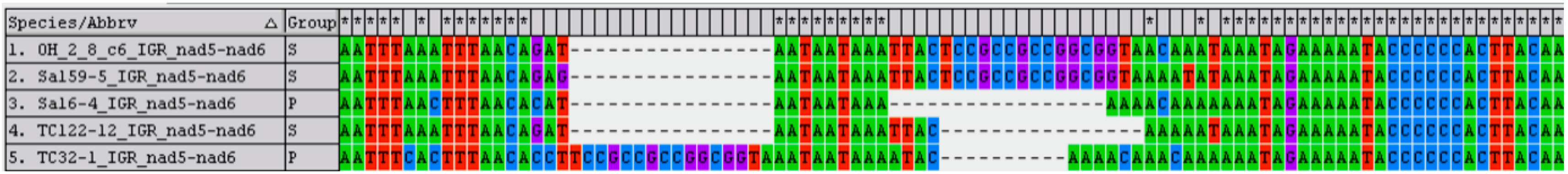

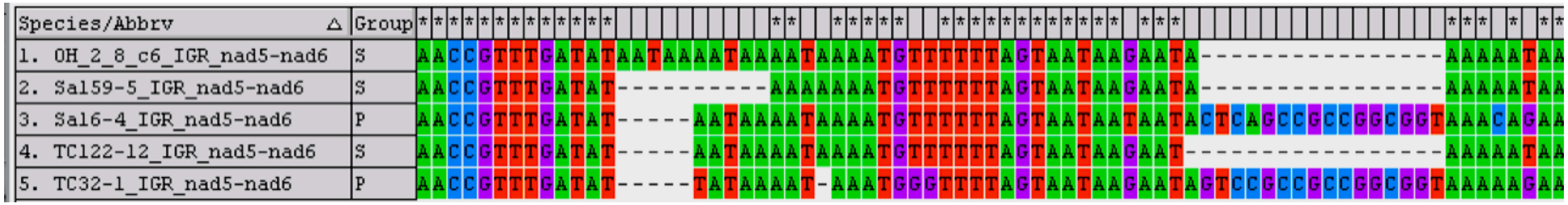

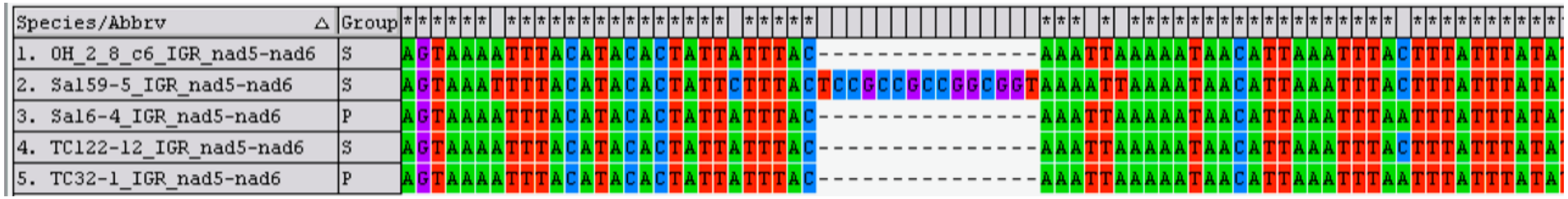

Species/Abbrv	Group	
1. OH_2_8_c6_IGR_nad5-nad6	S	AAATATTATAAATTATATTTT-------ATT-CTCCGCCGGCGGCGGCCAAATTTAAAAA
2. Sal59-5_IGR_nad5-nad6	S	AAATATTATAAATTATATTTTGT-----ATT-CTCCGCCGGCGGCGGCCAAATTTAAAAA
3. Sal6-4_IGR_nad5-nad6	P	AAATATTATAAATTATATTTTTT-----ATTACTCCGCCGCCGGCGGTCAAATTTAAACA
4. TC122-12_IGR_nad5-nad6	S	AAATATTATAAATTATATTTTTT-----ATT----------------ACAAATTTAAAAA
5. TC32-1_IGR_nad5-nad6	P	AAAGATTATAAATTATATTTTGTTTTTAATTAATCCGCCGCCAGCGGTAAACTTTAAAAA

Species/Abbrv	Group	
1. OH_2_8_c6_IGR_nad5-nad6	S	ATCCTTACTTAAAATTTA---------------GAGAATTATTTCCTATTAACTTTTTTATATT
2. Sal59-5_IGR_nad5-nad6	S	ATCCTTACTTAAAATTTA---------------GATAATTATTTCCTATTAACTTTTTTATATT
3. Sal6-4_IGR_nad5-nad6	P	ATCCTTACTGAAATTTTA---------------GATAATTATTTCCTATTAACTTTTTTATATC
4. TC122-12_IGR_nad5-nad6	S	ATCCTTACTTAAAATTTA---------------GATAATTATTTCCTATTAACTTTTTTATATT
5. TC32-1_IGR_nad5-nad6	P	ATCCTTACTTAAATGTTATCCGCCGGCGGCGGCTATAATTATTTCCTATTAACTTTTTTATATA

Species/Abbrv	Group	
1. OH_2_8_c6_IGR_nad5-nad6	S	ACCATTATTACTAATATT------TTTAGATTTATCAACCGCCGGCGGCCGAAATAAATAA--TTTTTTTTTGGTT
2. Sal59-5_IGR_nad5-nad6	S	ACCATTATTACTAAGATTATTATTTTTATATTTATCCGCCGCCGGCGGCCGAAATAAATCC---TTTTTTTTGTTA
3. Sal6-4_IGR_nad5-nad6	P	ACCATTATTACTAATATTATTATTTTTATATTTA----------------GAAAGAAATAC---TTTTTTTTGGTT
4. TC122-12_IGR_nad5-nad6	S	ACCATTATTACTAATATTTTTCTGTTTATATTTATCCGCCGCCGGCGGCCGAAATAAAATACTTTGTTTTTTGGTT
5. TC32-1_IGR_nad5-nad6	P	ACCATTATTACTAATATTATTATTTTTATATTTA----------------GAAAGAAATAC--TTTTTTTTTGGTT
